# Schneller Ausstieg oder bedachte Lockerung? Zur Zukunft von Kontaktbeschränkungen in der COVID-19-Krise

**DOI:** 10.1007/s10273-020-2634-2

**Published:** 2020-04-22

**Authors:** Sebastian Dullien, Alexander Herzog-Stein, Peter Hohlfeld, Sven Schreiber, Silke Tober

**Affiliations:** 1 Institut für Makroökonomie u. Konjunkturforschung, Hans-Böckler-Straße 39, 40476 Düsseldorf, Deutschland; 2 Ref. Makroökonomische Arbeitsmarktforschung, Institut für Makroökonomie u. Konjunkturforschung, Hans-Böckler-Straße 39, 40476 Düsseldorf, Deutschland; 3 Ref. Volkswirtschaftliche Gesamtrechnung, Institut für Makroökonomie u. Konjunkturforschung, Hans-Böckler-Straße 39, 40476 Düsseldorf, Deutschland; 4 Ref. Makroökonomische Grundsatzfragen, Institut für Makroökonomie u. Konjunkturforschung, Hans-Böckler-Straße 39, 40476 Düsseldorf, Deutschland; 5 Ref. Geldpolitik, Institut für Makroökonomie u. Konjunkturforschung, Hans-Böckler-Straße 39, 40476 Düsseldorf, Deutschland

**Keywords:** H00, I10, E60

## Abstract

Welche Bedeutung hat eine Lockerung der Kontaktbeschränkungen für die Erholung der deutschen Wirtschaft, und welche Schlussfolgerungen für das angemessene Niveau der Kontaktbeschränkungen sind in den kommenden Monaten zu ziehen? Um diese Fragen zu beantworten, wird abgeschätzt, welche Bedeutung die Maßnahmen zur Kontaktbeschränkung, die von Bund und Ländern seit Mitte März 2020 auf den Weg gebracht worden sind, für den derzeit zu beobachtenden Einbruch der Wirtschaftsaktivität in Deutschland tatsächlich haben. Zudem werden die verschiedenen plausiblen Szenarien zur Infektionsverbreitung und -eindämmung unter verschiedenen Optionen zur Lockerung der Kontaktbeschränkungen dargestellt.

## Abstract (en)

How important is the relaxation of contact restrictions for the recovery of the German economy and what conclusions can be drawn for the appropriate level of contact restrictions in the coming months? In considering these issues, an attempt will be made to assess what significance the contact restriction measures introduced by the federal and state governments since mid-March 2020 will actually have for the current slump in economic activity in Germany. In addition, the various plausible scenarios for the spread and containment of infection are presented with different options for easing contact restrictions.

Seit Beginn der Osterferien im April 2020 hat sich in Deutschland die Debatte zugespitzt, wie schnell und in welchem Maße die hier im Zuge der Bekämpfung der COVID-19-Pandemie verordneten Einschränkungen des öffentlichen Lebens und der Geschäftstätigkeit in Gastronomie, Tourismus, Freizeitbetrieben und Einzelhandel gelockert werden sollen. Die Hoffnung jener, die auf eine schnelle Öffnung drängen, ist, dass die negativen wirtschaftlichen Auswirkungen der Corona-Krise durch einen solchen Schritt begrenzt werden können. Dagegen stehen die Warnungen von Skeptikern, die ein Wiederaufflammen der Infektionen mit SARS-CoV-2 durch eine übereilte Öffnung fürchten.

### Kosten des Shutdowns versus Kosten der Öffnung

Seit Mitte März 2020 wurde eine Reihe von Kontaktbeschränkungen umgesetzt, die insbesondere Teile des Dienstleistungssektors hart getroffen haben. So wurden zunächst Schulen und Kindertagesstätten geschlossen. Seit dem 23. März sind zudem bundesweit große Teile des stationären Einzelhandels sowie Freizeiteinrichtungen, Restaurants und Cafés geschlossen. Touristische Übernachtungen sind untersagt. Die Bevölkerung ist angehalten, Kontakte auf ein Mindestmaß zu reduzieren, und – soweit möglich – von zu Hause aus zu arbeiten. Anders als etwa in Italien oder Spanien ist allerdings Produktionsbetrieben allgemein die Arbeit weiter erlaubt.

Um die direkten wirtschaftlichen Konsequenzen dieser Kontaktbeschränkungen von anderen globalen Folgen der Corona-Pandemie abzugrenzen, sollten die wirtschaftlichen Auswirkungen in Deutschland in drei überlappende Phasen unterteilt werden:

**Prof. Dr. Sebastian Dullien** lehrt Volkswirtschaftslehre an der Hochschule für Technik und Wirtschaft Berlin und ist Wissenschaftlicher Direktor des Instituts für Makroökonomie und Konjunkturforschung in der Hans-Böckler-Stiftung (IMK) in Düsseldorf.

**Prof. Alexander Herzog-Stein, Ph.D.**, ist Referatsleiter für Makroökonomische Arbeitsmarktforschung am IMK.

**Peter Hohlfeld**, Dipl.-Volkwirt, ist dort Referatsleiter für Volkswirtschaftliche Gesamtrechnung.

**PD Dr. Sven Schreiber** ist dort Referatsleiter für Makroökonomische Grundsatzfragen und Privatdozent an der Freien Universität Berlin.

**Dr. Silke Tober** ist Referatsleiterin für Geldpolitik am IMK.

- In Phase 1 standen der verringerte Absatz nach China und Unterbrechungen der Lieferketten infolge von Produktionseinschränkungen in China im Vordergrund.
- Phase 2 startete Mitte März 2020, als in Deutschland zur Eindämmung von SARS-CoV-2 Schulen wie auch Clubs, Restaurants und der nicht essenzielle Einzelhandel geschlossen wurden.
- Phase 3 ist durch den globalen Wirtschaftseinbruch gekennzeichnet, der für die stark exportabhängige deutsche Wirtschaft einen Rückgang der Produktion, der Investitionstätigkeit und in der Folge eine Schwächung des Konsums bedeutet.

In *Phase 1* hatte der Produktionseinbruch in China im Februar und März 2020 unmittelbare Folgen für die deutsche Wirtschaft. Durch die Produktionsunterbrechungen kam es zu Engpässen bei Vorprodukten insbesondere für die Automobil-, die Maschinenbau-, die Chemie- und die Elektronikindustrie, die einen Anteil an der Bruttowertschöpfung von 12 % haben. Mit zeitlicher Verzögerung folgte ein negativer Exportimpuls bei denselben Branchenschwerpunkten. In ähnlicher Weise waren die wichtigen europäischen Handelspartner Italien, Frankreich und Spanien betroffen, was wiederum negative Folgen für die deutsche Wirtschaft hatte.

*Phase 2* wirkte sich am stärksten auf die Wirtschaftsbereiche Handel, Gastgewerbe und Verkehr aus, die insgesamt ein Gewicht von 16,2 % an der Bruttowertschöpfung haben. Angesichts weitreichender Einschränkungen des Gastgewerbes dürfte dieses im zweiten Quartal 2020 nahezu zum Erliegen kommen, mit allerdings nur 1,5 % der Wirtschaftsleistung. Die im Zuge der Corona-Krise steigende Vorratshaltung bescherte dem Einzelhandel im Februar 2020 zunächst einen überdurchschnittlichen Umsatzanstieg: preisbereinigt gegenüber Januar 2020 im Bereich Lebensmittel, Getränke und Tabakwaren um 4,4 %; bei Apotheken, kosmetischen, pharmazeutischen und medizinischen Produkten um 4,1 %. Bei den Nicht-Lebensmitteln verzeichnete der Internethandel im Februar 2020 einen Umsatzsprung um 8 % gegenüber dem Vormonat. Im März dürfte sich diese Entwicklung noch etwas verstärkt fortgesetzt haben. Zudem gab es eine Verlagerung des Konsums von Restaurants hin zu direkten Lebensmittelkäufen.

Ab April 2020 dürfte bei hohen Lagerbeständen der Haushalte eine Normalisierung in diesen Bereichen stattfinden, während der Einzelhandel in anderen Bereichen einen Rückgang verzeichnen sollte. Hier wirken zwei gegenläufige Effekte: Einerseits haben die privaten Haushalte im Schnitt mehr Zeit für Konsumausgaben, andererseits kommt es trotz der wirtschaftspolitischen Stützungsmaßnahmen zu einer Verschlechterung der Einkommenssituation und einer zunehmenden Arbeitsplatzunsicherheit. Die Einschränkungen im Handel und im Verkehr führen insgesamt im zweiten Quartal auch hier zu einem deutlichen Einbruch, sodass für den gesamten Wirtschaftsbereich Handel, Gastgewerbe und Verkehr 2020 mit einem Rückgang um durchschnittlich mehr als 10 % zu rechnen ist (Dullien et al., 2020).

Das Produzierende Gewerbe ohne Bau (24,2 % der Bruttowertschöpfung) und der Bau (5,6 % der Bruttowertschöpfung) sind in Deutschland – anders als in anderen EU-Staaten – von staatlichen Beschränkungen nur insoweit betroffen, als hinreichende Schutzmaßnahmen für die Beschäftigten gewährleistet werden müssen. Nichtsdestotrotz dürfte die Produktion im Produzierenden Gewerbe ohne Bau vor allem wegen der außenwirtschaftlichen Faktoren um rund 12 % zurückgehen. Hier machen sich seit März 2020 verstärkt ausbleibende Vorprodukte aus anderen EU-Ländern wegen dortiger Produktionsunterbrechungen bemerkbar, auch weil innerhalb der EU Just-in-time-Lieferungen eine größere Rolle spielen als in den Lieferketten mit China. Der Bau dürfte trotz vereinzeltem Wegbleiben ausländischer Beschäftigter bei weiterhin guter Auftragslage zulegen. Die öffentlichen Dienstleister inklusive vieler Krankenhäuser haben einen Anteil von 18,8 % an der Bruttowertschöpfung und verzeichnen im Zuge der Pandemie eine deutlich steigende Aktivität.

Die jetzt einsetzende *Phase 3* ist eine Fortsetzung von Phase 1, wobei sich nun die chinesische Wirtschaft erholt, während die anderen Handelspartner Deutschlands den Corona-Schock durchleben. In dieser Phase entscheidet sich, ob die diversen massiven, nationalen Stützungsmaßnahmen eine sich verstärkende globale Abwärtsentwicklung der Weltwirtschaft verhindern können. Die Bedeutung der globalen Nachfrage zeigt sich an der mit 46,9 % hohen Exportquote der deutschen Wirtschaft, die in Schlüsselbereichen wie der Automobilindustrie mit 65 % und dem Maschinenbau mit 79 % sogar noch deutlich höher ist.

## Konjunkturprognose des IMK

Der jüngsten Prognose des Instituts für Makroökonomie und Konjunkturforschung (IMK) zufolge (Dullien et al., 2020) wird das Bruttoinlandsprodukt (BIP) in Deutschland im zweiten Quartal 2020 einen Einbruch um saison- und kalenderbereinigt 4,9 % gegenüber dem Vorquartal aufweisen und auch im dritten Quartal schrumpfen. Insgesamt dürfte damit das BIP im Jahresschnitt 2020 um 4,0 % unter dem Vorjahreswert liegen. Da das IMK für 2021 ein Wachstum von nur 2,4 % prognostiziert, wird das BIP im Jahresendquartal 2021 noch niedriger liegen als Ende 2019. Dieser Prognose liegt die Annahme zugrunde, dass die einschränkenden Maßnahmen ab Mai 2020 gelockert werden und sich die Weltwirtschaft im Laufe des Jahres zu erholen beginnt. Allerdings wird ebenfalls angenommen, dass die Ausnahmesituation, in der ein Abstandsgebot gilt, noch in das kommende Jahr hineinreicht. Insgesamt dürfte dabei deutlich weniger als die Hälfte des beobachteten Einbruchs der Wirtschaftsaktivität auf die seit Mitte März verhängten Kontaktbeschränkungen zurückzuführen sein; der größere Teil ist durch Produktions- und Absatzschwierigkeiten des Verarbeitenden Gewerbes verursacht. Daher wird sich die stark mit dem Ausland verflochtene deutsche Wirtschaft kurzfristig kaum ohne eine Erholung der Weltwirtschaft beleben können.

Würden die aktuell recht umfassenden physischen Kontaktbeschränkungen über einen dritten Monat hinweg ohne Lockerungen fortbestehen, so dürften die zusätzlichen ökonomischen Kosten allerdings überproportional höher ausfallen. Es dürfte dann zunehmend zu Insolvenzen kommen, und eine Nachfragebelebung könnte sich nicht unmittelbar in einer Wiederaufnahme der Wirtschaftstätigkeit niederschlagen. Die besonders betroffenen Bereiche der Gastronomie, der Hotels, des Nicht-Lebensmittelhandels sowie der Freizeit-, Sport- und Kulturdienstleister machen für sich genommen zwar nur rund 7 % der Bruttowertschöpfung aus. Von ihren Aktivitäten geht aber wiederum eine Nachfrage nach Dienstleistungen und Produkten anderer Bereiche aus, die damit ebenfalls weiterhin geschwächt würden. Insgesamt wäre zu erwarten, dass ein dritter Monat mit umfassenden Schließungen im Dienstleistungsbereich trotz der bestehenden wirtschaftspolitischen Stützungsmaßnahmen direkt und indirekt einen zusätzlichen Rückgang des deutschen BIP im Jahresdurchschnitt 2020 um rund 1 % bewirkt.

Noch schädlicher als eine länger anhaltende Kontaktbeschränkung wäre eine kurze Lockerung gefolgt von einer neuen, noch einmal längeren Phase von Beschränkungen mit geschlossenen Betrieben in Einzelhandel und Gastronomie. Weil in einem solchen Szenario die Gesamtdauer der Umsatzausfälle und Betriebsschließungen länger ausfallen würde, wäre auch mit größeren Rückgängen beim Bruttoinlandsprodukt zu rechnen.

Ein wichtiger Mechanismus, durch den eine länger anhaltende oder sich wiederholende Schließzeit bei Einzelhandel und Gastronomie zu überproportional höheren gesamtwirtschaftlichen Kosten führen kann, ist der Arbeitsmarkt. In seiner aktuellen Prognose geht das IMK davon aus, dass durch die zahlreichen Maßnahmen zur Stabilisierung von Wirtschaft und Beschäftigung analog zur Erfahrung während der Großen Rezession (siehe hierzu z. B. Herzog-Stein et al., 2018) in einem Best-case-Szenario die gesamtwirtschaftliche Beschäftigungssicherung abermals weitgehend gelingt, vor allem weil der Arbeitsmarkt durch die Nutzung von Kurzarbeit und anderen Instrumenten der internen Flexibilität in nie da gewesenem Ausmaß aufgefangen wird.

Dabei ist die erfolgreiche gesamtwirtschaftliche Beschäftigungssicherung stark davon abhängig, dass für die ökonomischen Akteure durch einen realistischen Plan einer schrittweisen Lockerung der Kontaktbeschränkungen die Krisendauer abschätzbar, und die daran anschließende wirtschaftliche Erholung vorstellbar sind. Mit jedem zusätzlichen Tag umfassender Einschränkungen des öffentlichen Lebens in Deutschland über Anfang Mai 2020 hinaus steigt das Risiko, dass die Betriebe und die Beschäftigten die massiven Anstrengungen für eine erfolgreiche Beschäftigungssicherung nicht länger durchhalten und zunehmend Beschäftigte entlassen werden. Umfangreiche Beschäftigungsverluste hätten dann massive negative Auswirkungen auf die gesamtwirtschaftliche Nachfrage und würden so eine schnelle Erholung unwahrscheinlich machen. Im Extremfall würden viele Unternehmen, Ladengeschäfte und Restaurants dauerhaft vom Markt verschwinden. Der Verlust eines erheblichen Teils des gesamtwirtschaftlichen Kapitalstocks brächte die Gefahr einer negativen Pfadabhängigkeit der Arbeitsmarktentwicklung mit sich. Eine gedämpfte Beschäftigungsentwicklung, eine Rückkehr sowie Verfestigung der Massenarbeitslosigkeit und negative Hystereseeffekte[1] wären mittel- und langfristig zu befürchten.

Ein langes Anhalten der aktuellen, umfassenden Kontaktbeschränkungen könnte auch über den Bildungssektor größere volkswirtschaftliche Kosten entstehen lassen. Zwar betrifft die Schließung der Schulen nicht das aktuelle BIP, sie schränkt jedoch erstens die Arbeitsfähigkeit erwerbstätiger Eltern ein und wirkt zweitens negativ auf die Humankapitalbildung. In der akuten Krisensituation, in der viele Betriebe ohnehin Kurzarbeit angemeldet haben, ist der Ausfall von berufstätigen Eltern zwar noch zweitrangig, zumal die Zahl betroffener Eltern überschaubar ist.[2] Über eine längere Zeit dürfte es allerdings schwieriger werden, den Ausfall der Kinderbetreuung zu kompensieren. Auch hier dürften die negativen Effekte mit anhaltender Dauer überproportional sein.

Gleiches gilt für die Humankapitalbildung. Die meisten Schulen in Deutschland sind nicht ausreichend vorbereitet, ihre Schülerinnen und Schüler digital zu unterrichten. Gerade in sozialen Brennpunkten wäre eine Ausstattung mit Tablets und anderen Geräten mit Internetzugang erforderlich, damit Lernende im Fall einer erneuten Schließung oder Quarantäne in der Lage sind, das bereits existierende Lernangebot zu nutzen und Feedback von Lehrkräften zu erhalten. Ein Unterrichtsausfall von drei Wochen dürfte hier zwar insgesamt verkraftbar sein. Sollten sich die Schulschließungen allerdings deutlich länger hinziehen, ist mit zunehmenden Lücken insbesondere bei Kindern aus sozial schwachen Familien zu rechnen.

1 Zu den möglichen Ursachen von Hysterese bei der Arbeitslosigkeit siehe Røed (1997).

2 Rund 17 % der 45 Mio. erwerbstätigen Personen in Deutschland lebten 2018 in Doppelverdienerhaushalten mit Kindern im Alter unter 13 Jahren oder waren alleinerziehend.

## Dynamik der Infektionsverbreitung

Zur Bewertung der Kosten und des Nutzens der Kontaktbeschränkungen ist neben den wirtschaftlichen Wirkungskanälen auch ein Verständnis des Verlaufs der Infektions-, Erkrankungs- und Todeszahlen in verschiedenen Szenarien notwendig. In der Epidemiologie werden für eine Grobabschätzung in der Regel sogenannte Susceptible-Exposed-Infectious-Recovered-Modelle (SEIR-Modelle) verwendet.[3] Aus der Literatur zur SARS-CoV-2-Ausbreitung und IMK-interner Simulationen mit diesen Modellen mit in aktuellen Studien geschätzten Modellparametern[4] sind folgende Schlussfolgerungen für den Infektionsverlauf zu ziehen:

- Ein einfaches Durchlaufenlassen der Infektion (hypothetisch ohne die Kontaktreduzierungen seit Mitte März) führt schnell zu sehr vielen Infizierten, vielen Patienten in intensivmedizinischer Behandlung und einer fast sicheren Überlastung des Gesundheitssystems. Je nach Annahme zur Basisreproduktionsrate $R_0$, die angibt, wie viele Menschen im Durchschnitt von jeder infizierten Person angesteckt werden, wäre der Höhepunkt irgendwann im (Früh-)Sommer 2020 erreicht. Dabei könnte es mehrere Hunderttausend Tote geben; unter gängigen Annahmen zur Sterblichkeit bei unverändertem Verhalten wären zum Höhepunkt mit täglich mehreren Zehntausend Toten zu rechnen. Da bei Überlastung des Gesundheitssystems die Sterblichkeit noch einmal deutlich höher ausfallen könnte und es auch zu einer massiven Gefährdung des Personals im Gesundheitssektor kommen würde, sollte dieses Szenario unbedingt vermieden werden.

- Ein einfaches Aufheben der Kontaktbeschränkungen und eine Rückkehr zur Verbreitungsrate $R_0$ von vor Einführung der Maßnahmen bedeutet eine Rückkehr zu exponentiellem Infektionswachstum innerhalb von wenigen Wochen. In einem solchen Szenario wäre gegenüber dem einfachen Durchlaufenlassen der Infektion nur wenig Zeit gewonnen; es erscheint allerdings unrealistisch, innerhalb dieser kurzen Zeit die Kapazitäten im Gesundheitssystem ausreichend erweitern zu können, um die zu erwartende Infektionswelle zu bewältigen. Damit hätte eine solche Strategie kaum einen Vorteil: Die ökonomischen und sozialen Kosten der Kontaktbeschränkung wären angefallen, ohne einen großen Nutzen zu erreichen.

- Auch eine Strategie, die zunächst zu den Vor-Krisen-Verhaltensweisen zurückkehrt und auf einen neuen Anstieg der Infektionen stattdessen mit einer Wiedereinführung der Beschränkungen reagiert, würde bis ins Jahr 2021 hinein immer wieder restriktive Phasen wie in der zweiten März-Hälfte erforderlich machen. Die Zeiten mit Beschränkungen dürften hier sogar überwiegen. Die ökonomischen Kosten des teilweisen Shutdowns würden noch drastisch erhöht. Dies erscheint deshalb ebenfalls nicht als eine sinnvolle Strategie.

Da es gleichzeitig angesichts der engen Verflechtungen der deutschen Wirtschaft mit den Volkswirtschaften Europas und der Welt nicht realistisch erscheint, Neuinfektionen völlig auszuschließen, bleibt als einzige – auch ökonomisch – sinnvolle Strategie, die Reproduktionsrate nachhaltig und bis zur Verfügbarkeit eines Impfstoffs niedrig und möglichst unter dem Wert von 1 zu halten. Während derzeit umstritten ist, welche Maßnahmen die Reproduktionsrate wie stark senken und welchen Beitrag jenseits verordneter Maßnahmen Verhaltensänderungen der Bevölkerung spielen, ist eines klar: Eine einfache Rückkehr zu dem Leben und Wirtschaften wie vor Ausbruch der Pandemie wird es in den kommenden Monaten höchstwahrscheinlich nicht geben.

## Politikschlussfolgerungen: schneller Ausstieg oder bedachte Lockerung?

Aus diesen Überlegungen folgt, dass die aktuellen Kontaktbeschränkungen ökonomisch zwar sehr teuer sind, aber die (langfristigen) Kosten einer zu frühzeitigen Aufhebung noch größer wären. Eine vorschnelle Aufhebung würde riskieren, dass aufgrund eines neuen Aufflammens der Infektionen innerhalb kürzester Zeit erneut harte Kontaktbeschränkungen für einen längeren Zeitraum verhängt werden müssten, was die ökonomischen Gesamtkosten drastisch in die Höhe treiben würde. Die Kontaktbeschränkungen nachhaltig zu lockern ist also wichtiger, als sie schnell aufzuheben.

Für die deutsche Wirtschaft ist dabei eine Erholung des Verarbeitenden Gewerbes mindestens ebenso wichtig wie eine Erholung des stationären Einzelhandels und der Gastronomie. Eine vorschnelle Aufhebung von Kontaktbeschränkungen und Abstandsgebot, die zu einer Verschärfung der Infektionen und am Ende zu Produktionsstörungen führt, wie sie in Spanien und Italien erlebt wurden, wäre kontraproduktiv.

Für die Lockerung der Kontaktbeschränkungen selber lassen sich drei grundsätzliche Schlussfolgerungen ableiten:

1. Lockerungen sollten schrittweise und vorsichtig erfolgen. Dabei sollten jene Lockerungen der Kontaktbeschränkungen zunächst durchgeführt werden, die möglichst geringe Ansteckungsgefahren (und insbesondere geringe Gefahren für Risikogruppen) mit sich

3 Siehe exemplarisch etwa an der Heiden und Buchholz (2020) oder für einen Überblick Hethcote (2000). Für feinere Abschätzungen werden diese Modelle oft um Netzwerkaspekte oder Bevölkerungsgruppen erweitert.

4 So wird üblicherweise von einer Basisreproduktionszahl $R_0$ von SARS-CoV-2 in der Größenordnung zwischen 2,4 und 3,3 ausgegangen sowie einem Fall-Verstorbenen-Anteil von um 1 %. Vgl. Robert Koch-Institut (2020).

bringen und deren Aufrechterhalten zudem besonders hohe ökonomische und soziale Kosten verursachen.

2. Es sollte systematischer zusammengetragen und kommuniziert werden, über welche spezifischen und situationsbedingten Maßnahmen wie etwa Schutzkleidung, Barrieren und Plexiglasscheiben oder Zugangsbeschränkungen für Lokalitäten die Ansteckungsgefahren so verringert werden, dass wirtschaftliche Aktivitäten wieder möglich werden.

3. Alle notwendigen Ressourcen sollten in Möglichkeiten des datenschutzkonformen Trackings und Nachverfolgens von Infektionsketten gesteckt werden.

Selbstverständlich ist, dass dabei das Design der konkreten Ausstiegsschritte sinnvoll nur interdisziplinär erarbeitet werden kann.

Insbesondere beim Tracking und dem Nachverfolgen von Infektionsketten sollte die EU-Perspektive gefördert werden. Wichtig ist hier, die nationalen Systeme kompatibel zueinander zu halten, um bald wieder den möglichst ungehinderten Grenzverkehr von Arbeitskräften zu ermöglichen, ohne neue Infektionsketten zu riskieren. Außerdem ist die grenzüberschreitende Zusammenarbeit wichtig, um Lieferketten innerhalb der EU für die nächsten Monate stabil zu halten. Die Gefahr, dass ein neuer Infektionsausbruch in einem EU-Mitgliedsland die Lieferung von Vorprodukten erneut unterbricht, ist insbesondere für die stark vernetzte deutsche Volkswirtschaft relevant.

Angesichts der hohen ökonomischen Kosten der derzeit bestehenden Kontaktbeschränkungen sollte bei Ausgaben und Investitionen für den Infektionsschutz nicht gespart werden. In den allermeisten Fällen dürften die notwendigen Veränderungen an Lokalitäten oder Geschäftsabläufen, die notwendigen Investitionen in Infrastruktur sowie die Kosten flächendeckender Tests weit geringer sein als die Alternativkosten einer langen Fortsetzung sehr restriktiver Kontaktbeschränkungen.

### Maßnahmen zur Eindämmung der Corona-Krise

Konkret ließen sich aus diesen Schlussfolgerungen folgende Maßnahmen ableiten:

- Kapazitäten für Tests und zur Nachverfolgung von Infektionsketten sollten schnellstmöglich ausgebaut werden; die Nutzung von Handy-Apps sollte dabei geprüft werden, allerdings aufgrund des Datenschutzes ohne zentrale Speicherung von Kontakt- oder Bewegungsprofilen und zeitlich begrenzt. Denkbar wäre auch, den Zugang etwa zu Restaurants zunächst für jene zu lockern, die bereit sind, ihre Kontaktdaten zu hinterlassen.

- In allen Unternehmen sollten mit Nachdruck und kooperativ zwischen Geschäftsführung und Beschäftigten Maßnahmen für einen wirkungsvollen Infektionsschutz organisiert werden.

- In Einzelhandel, Gastronomie und derzeit geschlossenen Dienstleistern mit Kundenkontakt, wie Friseursalons, müsste geprüft werden, wie über den Einbau von Virusbarrieren, die Einhaltung des Abstandsgebots und die Nutzung von Schutzkleidung ein Betrieb mit möglichst begrenzter Infektionsgefahr möglich ist.

- Für den Bereich der Schulen und Kitas sollte – soweit möglich – durch Separation und Kohortierung bestimmter Gruppen zumindest ein teilweiser Betrieb wieder aufgenommen werden (etwa: Unterricht nur noch jeden zweiten Tag bei klarer räumlicher Trennung von Klassen). Soweit mangelnde Infrastruktur wie knappe sanitäre Einrichtungen dem im Wege stehen, sollten schnell und unbürokratisch Provisorien geschaffen werden.

- Soweit Mund-Nasen-Schutz verfügbar ist, sollte überprüft werden, inwieweit eine generelle Pflicht zum Tragen dieser einfachen Masken auf öffentlichen Wegen die Infektionsverbreitung begrenzen könnte.

- In den Fällen, in denen es akute Beschränkungen der verfügbaren Ressourcen gibt (etwa bei Atemmasken), ist ein schnelles Hochfahren der heimischen Produktion sinnvoll und sollte von der Regierung durch Großbestellungen und langjährige Lieferverträge gefördert werden. In der Übergangsphase sollten Maßnahmen wie etwa die Einführung von Maskenpflicht so abgestimmt werden, dass zunächst Bereiche mit größerer Bedeutung für eine Begrenzung der Verbreitung des Coronavirus (also Gesundheits- oder Pflegebereich) ausreichend versorgt sind.

- Sinnvoll wäre ein regional abgestuftes Vorgehen bei der Lockerung wie von Bofinger (2020) vorgeschlagen. So könnten Landkreise mit niedrigen Infektionszahlen oder -dynamik oder weit vorangeschrittenen Maßnahmen für Infektionsschutz den Einzelhandel, Schulen, Kitas und Gastronomie schrittweise vor anderen Regionen wieder öffnen. Wichtig wäre hier allerdings, streng durchzusetzen, dass nur Menschen mit Wohnsitz in dem entsprechenden Landkreis dort bedient werden, um neue interregionale Infektionsketten zu vermeiden. Auch in diesen Gebieten sollte das Abstandsgebot weiter gelten. Eine solche vorgezogene Lockerung in einzelnen Regionen könnte nicht nur wichtige Erkenntnisse über wirkungsvolle Einzelmaßnahmen der Infektionsvermeidung geben, sondern auch die Zahl der Insolvenzen verringern.

- Ebenfalls sollte darauf vorbereitet werden, dass eine erneute, graduelle Verschärfung der Kontaktbeschränkungen notwendig werden könnte. Hier sollte insbesondere an den Schulen – auch nach einer (teilweisen) Öffnung – die digitale Infrastruktur verbessert werden. Zentral ist hier unter anderem die Versorgung wirtschaftlich benachteiligter Kinder und Jugendlicher mit Laptops oder Tablets sowie den dafür notwendigen Internetzugängen.

Angesichts der beschriebenen Infektionsdynamik und der Gefahr eines Wiederaufflammens der Pandemie scheint auch ökonomisch das Vorsichtsprinzip bei der Lockerung der Kontaktbeschränkungen angebracht: Die Öffnung sollte nach und nach erfolgen und die notwendigen Maßnahmen zur Infektionsbegrenzung auf jeden Fall vorher auf den Weg gebracht werden.

Für eine rechtzeitige Umsetzung ist allerdings eine schnelle und klare Kommunikation dieses Verfahrens notwendig, sodass etwa Einzelhändler die Zeiten der verordneten Schließung zur Vorbereitung der Wiedereröffnung nutzen können. Bislang gibt es in der Kommunikation einer Ausstiegs- oder Lockerungsstrategie der Regierung noch Schwächen. So ist bislang nicht klar kommuniziert worden, unter welchen Bedingungen eine Lockerung zu erwarten ist. Über vorbereitende Maßnahmen einer Lockerung der Kontaktbeschränkungen gibt es keinerlei Aussagen. Ebenfalls wäre eine klare Aussage der Regierung wünschenswert, dass es in den kommenden Monaten keine vollständige Rückkehr zu dem Status quo vor der Krise geben wird und bestimmte Bereiche wie Großveranstaltungen sich auf länger bestehende Beschränkungen einstellen müssen. Eine klare Kommunikation könnte in der aktuellen labilen Lage nicht nur die Erwartungen der Unternehmen auf Absatzerholungen stabilisieren, sondern auch zu einer beschleunigten Umsetzung notwendiger Maßnahmen gegen die Infektionsverbreitung führen.

Darüber hinaus ergibt unsere ökonomische Analyse, dass die Lockerung der Kontaktbeschränkungen zwar eine notwendige, aber keine hinreichende Bedingung dafür ist, dass die deutsche Wirtschaft möglichst unbeschadet durch die Corona-Krise kommt. Jenseits des besonders im Fokus stehenden Dienstleistungs- und Einzelhandelsbereichs besteht die Gefahr, dass durch negative Rückkopplungen im internationalen Handel in den kommenden Monaten auch das Verarbeitende Gewerbe von der Nachfrageseite noch einmal unter Druck gerät. Wichtig wäre, alles zu tun, um den europäischen Binnenmarkt zu stabilisieren. Hier wäre vor allem wichtig, ein Signal der Solidarität an die EU-Partner zu senden und eine weiter destabilisierende Staatsschuldenkrise im Euroraum zu begrenzen. Die von Bofinger et al. (2020) vorgeschlagenen Corona-Bonds wären hier ein sinnvoller Schritt. Außerdem sollte ein – nach Möglichkeit – international abgestimmtes Konjunkturpaket auf den Weg gebracht werden, um einen Beitrag Deutschlands zur Stabilisierung der Weltwirtschaft zu liefern und die Schwäche der Exportnachfrage auszugleichen.
